# Accounting for health in climate change policies: a case study of Fiji

**DOI:** 10.3402/gha.v7.23550

**Published:** 2014-05-15

**Authors:** Georgina Morrow, Kathryn Bowen

**Affiliations:** 1 Institute of Tropical Medicine and International Health, Charité – Universitätsmedizin Berlin, Berlin, Germany; 2 National Centre for Epidemiology and Population Health, Australian National University, Canberra, Australia

**Keywords:** climate change, health, Fiji, policy, policy analysis, pacific islands

## Abstract

**Background:**

Climate change is expected to affect the health of most populations in the coming decades, having the greatest impact on the poorest and most disadvantaged people in the world. The Pacific islands, including Fiji, are particularly vulnerable to the effects of climate change.

**Objective:**

The three major health impacts of climate change in Fiji explored in this study were dengue fever, diarrhoeal disease, and malnutrition, as they each pose a significant threat to human health. The aim of this study was to investigate to what extent the Fiji National Climate Change Policy, and a selection of relevant sectoral policies, account for these human health effects of climate change.

**Design:**

The study employed a three-pronged policy analysis to evaluate: 1) the content of the Fijian National Climate Change Policy and to what extent health was incorporated within this; 2) the context within which the policy was developed; 3) the relevant processes; and 4) the actors involved. A selection of relevant sectoral policies were also analysed to assess the extent to which these included climate change and health considerations.

**Results:**

The policy analysis showed that these three health impacts of climate change were only considered to a minor extent, and often indirectly, in both the Fiji National Climate Change Policy and the corresponding National Climate Change Adaptation Strategy, as well as the Public Health Act. Furthermore, supporting documents in relevant sectors including water and agriculture made no mention of climate change and health impacts.

**Conclusions:**

The projected health impacts of climate change should be considered as part of reviewing the Fiji National Climate Change Policy and National Climate Change Adaptation Strategy, and the Public Health Act. In the interest of public health, this should include strategies for combating dengue fever, malnutrition, and water-borne disease. Related sectoral policies in water and agriculture should also be revised to consider climate change and its impact on human health. Approaches to include health aspects of climate change within sectoral and climate change specific policies should be encouraged, via a number of mechanisms, such as the Health in All Policies approach. Future research could support the Fiji health sector in developing climate change and health programmes.

Climate change has been identified as one of the greatest challenges for humankind and responding to the impacts of climate change is a key priority for the health sector (1–8). The health of most populations is expected to be affected by climate change, with the greatest impact predicted to be on the poorest and most disadvantaged people in the world (1, 9). Climate change is projected to result in an increase in the intensity and/or frequency of extreme weather events, such as cyclones, and seasonal changes which will affect Fiji by changing rainfall patterns (10, 11).

The Pacific Islands, including Fiji, are highly vulnerable to the adverse health impacts of climate change, due to i) being prone to natural disasters, ii) their underlying levels of disease, and iii) their often poorly equipped health services (12). Hence, it is important to understand the extent to which public policies within Fiji account for the health impacts of climate change.

All public policies can influence health by forming healthy environments (13). Many sectoral policies, such as water and agriculture, have important consequences on population health. For example, the ‘Health in All Policies’ (HiAP) is one method that takes a broad cross-sectoral view of the place of health within public policy. The goal of HiAP is to improve population health by reforming public policy making throughout all sectors, via the inclusion of health considerations within different sectoral policies (13). The health impacts of climate change are a potent example of the importance of a cross-sectoral approach; given that the majority of the health impacts of climate change will arise from other sectors (e.g. water and agriculture) (14), it is crucial that a holistic framing of health is used to ensure that relevant sectors understand and take action on health impacts.

This study aims to analyse the extent to which the Fiji National Climate Change Policy (NCCP), and a selection of other relevant policies, accounts for the human health impacts of climate change. Using a policy analysis technique, this study focuses on the content of the NCCP and relevant documents (15, 16). A background analysis of context, process and culture give insight into factors influencing the NCCP content.

## Background

### Climate change projections for Fiji

Pacific Island countries are particularly vulnerable to the impacts of global climate change and are among the most susceptible to natural disasters (12). This is largely due to their high potential exposure to extreme weather events and low adaptive capacity (17).

In Fiji, it is projected that the average ambient temperature as well as the intensity and frequency of days of extreme heat and extreme rainfall will increase (11). Moreover, ocean temperature, ocean acidification and sea levels will continue to rise (11). It is projected that up to 14% of coastal land will be lost due to a sea-level rise by 2050 (18).

A moderately confident projection exists for an increase in the frequency of cyclones, while rainfall, although maintaining a similar annual mean, will increase in the wet season and decrease in the dry season (11). This will exacerbate an existing vulnerability to floods and drought (19).

### Predicted health effects of climate change

These changes in climate are expected to affect human health worldwide both directly and indirectly (2). The direct effects relate primarily to extreme weather events (20, 21). It is projected that there will be an increase in disease, injury and even death caused by heat waves, cyclones, floods, and drought (10).

Climate change can affect human health indirectly through its impact on important sectors, such as agriculture and water (21). These indirect causes are expected to have the greatest impact (2). For example, food yield will be affected by destruction of crops during extreme events, soil erosion, and salinity from flooding, poor crop harvests during drought, and contamination of groundwater owing to sea-level rise and brackish water (4, 22). All of these circumstances will have implications for the public health of communities.

In Fiji, clear correlations exist between climate and diarrhoeal outbreaks and it is projected that the risk of contracting diarrhoea will increase (23), as changing patterns of rainfall can contribute to the contamination of surface and drinking water (3). Infrastructure breakdown in extreme weather events (in particular for water and sewage) is also likely to play a role in an increase of diarrhoeal outbreaks (4, 24).

It is expected that changes in precipitation patterns and humidity will increase the endemic range of some vectors (25, 18). In Fiji, the major vector of concern is *Aedes aegypti*, the mosquito that transmits dengue fever. Although climate change is a contributing factor to the incidence of vector-borne disease, it is not the only one. Socio-economic factors, along with the quality of health care and public health infrastructure also contribute (20, 26). For example, people's access to good water infrastructure and waste disposal (wet containers are common breeding grounds for *Aedes aegypti*) are often income-related (26). Disease prevention programmes and vector control efforts can reduce the health risks of dengue fever (20).

With human health in Fiji expected to be affected by climate change, it is of vital importance that these adverse health impacts are addressed by the NCCP and supporting documents. In line with recent Fijian vulnerability assessments (18, 27), three of the many priority climate-sensitive health risks in Fiji are an increased morbidity and mortality of dengue fever, diarrhoeal disease and malnutrition (in this case malnutrition relates to under nutrition rather than over nutrition). These are, therefore, the major foci of this policy analysis.

## Method

### Policy analysis

To adequately understand the NCCP and National Climate Change Adaptation Strategy (NCCAS), this study utilised the policy analysis framework developed by Walt and Gilson (28). As opposed to traditional techniques in which the content was the sole element, this framework revolutionised policy analysis by broadening its scope. This method was selected, as it not only explores the *content* of policy reform, but also the *context* within which the policy is developed and the *processes* involved. Integrated in this three-pronged framework are the *actors* involved. This framework was used by analysing each of the four aforementioned aspects of the policy separately, then synthesising these elements to form an integrated understanding and detailed discussion of the NCCP and NCCAS.

In the case of climate change policy in Fiji (and more generally), it is important to consider the background factors influencing the content of the policy, as understanding of the context, process, and actors involved can help explain why certain policy outcomes emerge (28). As such, these background factors set the scene for the content analysis.

### Background analysis – context, process, and actors

In terms of the context analysis, the heuristic model provided by Leichter (29) was used in order to gain a deeper understanding of major contextual factors influencing the policy development, including situational, structural, and cultural.

1. *Structural* factors are relatively stable elements of the polity and society.
2. *Situational* factors are temporary circumstance that impact policy making.
3. *Cultural* factors are value systems operating within a community.

The structural factors considered were geography, demographics, urbanisation, and poverty, as these are frequently discussed in the literature as both highly relevant not only to the Fijian climate change context, but also globally (1, 9, 30).

Globalisation and brain-drain were chosen as relevant situational factors for similar reasons, while the coup could not be ignored as a major political circumstance impacting policy in Fiji. Finally, the cultural diversity in Fiji was seen as particularly unique and important to consider.

The processes involved in the NCCP were analysed to help to understand how it was developed and who influenced it. The final element of this policy analysis was an assessment of climate change documents relevant to Fiji. In addition, the actors involved (both directly and indirectly) in policy making were considered, as they are influenced by the context within which they live and work (28).

### Content analysis

The NCCP and other relevant documents were analysed in order to identify the extent to which the three major predicted health effects were accounted for in the policies. ‘Extent’ was defined as the existence or mention of a given health effect and the frequency with which this health effect was included in the policy. Important supporting documents were also analysed for the extent to which climate change effects were factored into the sectoral policies and strategies.

### Documents

The key Fijian policy and strategy documents analysed were:

1. National Climate Change Policy (NCCP)
2. National Climate Change Adaptation Strategy (NCCAS)

Supporting documents analysed were:

1. The Public Health Act (31)
2. The National Environmental Health Action Plan (NEHAP) (32)
3. Fiji National Food & Nutrition Policy (33)
4. Rural Land Use Policy for Fiji (34)
5. National Water Resources Policy for the Fiji Islands (Draft Water Policy) (35)

### Accessing documents

Accessing literature, policies, and other documents for the policy analysis involved two main steps: i) literature search and review of peer-reviewed publications, using the databases: PubMed, MEDLINE, Embase, and GreenLine; and ii) review of policy documents of national government, regional organisations, and stakeholders (publically available online). Key words for both searches included climate change, health, Pacific, Fiji, and policy.

## Results

### Background analysis: process, context, and actors

#### Process

The NCCP Framework, outlining the government's position on climate change, was endorsed by cabinet in 2007. It directed the responsibilities of each relevant stakeholder. The framework was reviewed in 2011 in light of current research and climate predictions in the region (15).

Passed by cabinet on 19 January 2012, the NCCP was developed through what was described in the policy itself as an extensive consultative process with stakeholders in government, non-governmental organisations (NGOs), academia, community representatives, and the private sector (15). Led by the Climate Change Unit (CCU), the policy development was guided by the NCCP Taskforce and the internal policy committee, before being approved by the National Climate Change Country Team (NCCCT) (15). The NCCCT includes representatives of various government ministries, NGOs, development partners, regional agencies, and UN agencies (36).

Monitoring the implementation of the NCCP, the CCU formulates quarterly progress reports and submits an annual report to the NCCCT and the National Environment Council. The policy will be fully reviewed in 2017, 5 years after its adoption (15).

#### Context: structural factors

*Geography and demographics*. Fiji is located in the Pacific Ocean and consists of around 330 islands, of which about one third are inhabited (37). The median age of the population is 27.6 years, with the overall male:female ratio being 1.03:1 (38). The majority of the population live along coastal flood plains (37) and are thereby exposed to extreme weather events.

*Urbanisation*. Over half the Fijian population currently live in urban areas (38) and the trend of rural to urban migration is anticipated to continue (18). This could result in further densification of urban infrastructure, and sprawling of urban and peri-urban areas along the coast contributing to a rise in structurally inadequate housing (18). Rapid and unplanned urbanisation could exacerbate nutrition-related disease through food insecurity and water-borne disease via poor access to clean water (39, 40).

*Poverty*. A range of studies have placed the likely number of households living below the poverty line to be between 12 and 20%, with the urban fringe areas of the nation's capital, Suva, reaching up to 35% (18). Poor populations are among those most vulnerable to the adverse effects of climate change, as they have the fewest resources and least ability to adapt (1).

#### Context: situational factors

*Globalisation*. Food security is one of the three major expected health effects of climate change facing Fiji. A situational factor adding to this threat is that of globalisation, which can affect food security threefold. Firstly, by an increased dependence on imported staple foods, secondly, through international agreements and trade regulations, and thirdly, through international economic and political fluctuations (39).

*Coup*. The current Fijian leadership situation is complex and has contributed to national priorities, including the initiation of the NCCP. On 5 December 2006, Commodore Voreqe Bainimarama led a military coup, ousting the elected Fijian government and replacing it with a so-called ‘interim’ government (41).

Internationally the coup was viewed as being illegal, and thus in 2009, Fiji was suspended from both the Pacific Island Forum and the British Commonwealth (41). Australia, New Zealand, and the European Union (EU) enforced travel bans on all people involved in the coup or employed by the interim government (42). New Zealand also suspended training programmes for the Fiji public sector and froze a seasonal workers scheme (42). The largest purchaser of Fijian sugar is the EU, which continues to exert pressure on Fiji towards democratic elections (which have not occurred since the coup in 2006) in order to continue trade. By law, the United States cannot supply aid to countries where a coup has taken place (42). These international sanctions affect the implementation of climate change adaptation projects, which rely partly on external funding.

*Brain-drain*. Regarding health treatment and prioritisation, Fiji is faced with the situation of so-called brain-drain, particularly since the 2006 coup (42). Brain-drain describes a phenomenon, whereby large numbers of (in this case) Fijian doctors and nurses continue to emigrate to larger neighbouring countries, such as Australia and New Zealand (40). This exerts pressure on the local health system, which has to cope with understaffing, for example, one physician per 2,000 people (43). Although this is not considered in the NCCP, this is an important contextual factor when addressing public health initiatives related to climate change.

#### Context: cultural factors

Although the Fijian government formally operates at national and district levels, a traditional customary structure simultaneously operates in all levels of society. This system is formalised through the Ministry of iTaukei Affairs, responsible for the welfare and governance of the original and native settlers of Fiji (16, 44).

The 2007 national census reflected the ethnic diversity in Fiji: ethno-Fijian 57.3%, ethno-Indian 37.6%, Rotuman 1.2%, other 3.9% (38). Historically, there has been tension between the two major ethnic groups (ethno-Fijian and ethno-Indian), sometimes resulting in political conflict (42). Currently, internal support for the coup and the interim government is divided (42), which could provide challenges for implementing policies, such as the NCCP.

#### Actors, stakeholders, and documents

*National stakeholders with direct responsibly for climate change policy*. As well as coordinating climate change programmes in Fiji, the CCU delivers the NCCP. Originally established within the Department of Environment in 2009, the CCU moved to the Ministry of Foreign Affairs and International Cooperation in 2011. This relocation was described as a strategic move for increasing support for climate change programmes in Fiji (15). It is also relevant to note that one of the NCCP goals is to increase access to global financing for climate change adaptation projects (15).

The NCCCT, which serves the National Environmental Council, provides guidance to the CCU on climate change issues and is the major platform for reporting on climate change projects. The NEC, on the contrary, facilitates the creation of environment-related policies and strategies (16).

*Other governmental ministries*. Sitting within the Ministry of Provincial Development and National Disaster Management is the National Disaster Management Office, which formulates disaster management policies and provides information on all disaster-related matters (15).

*Other national stakeholders*. It is important to note that citizens of Fiji and the private sector play a role in implementing climate change projects and working with the government to achieve its goals.

*NGOs and funding bodies*. Many organisations provide financial and project support to Fiji's climate change adaptation and mitigation efforts. These include, but are not limited to (45):

1. Deutsche Gesellschaft für Internationale Zusammenarbeit (GIZ), which co-funded the development of the NCCP
2. Secretariat of the Pacific Community (SPC), which co-funded the development of the NCCP
3. The World Bank
4. Asian Development Bank
5. Australian Agency for International Development
6. European Union
7. United Nations Development Programme
8. United States Agency for International Development

*Academic institutions*. The University of the South Pacific (USP) provides research and academic knowledge in Fiji and the region. Furthermore, the USP's Pacific Centre for Environment and Sustainable Development implements a Global Climate Change Alliance project, building the capacity of Pacific countries to adapt to impacts of climate change (46).

**Table:** Supporting documents

Title	Relevant Organisation	Purpose
*Global*		
Hyogo Framework (47)	United Nations Office for Disaster Risk Reduction	International strategy for disaster risk reduction
Kyoto Protocol (48)	United Nations Framework Convention on Climate Change	Sets international emissions targets
AOSIS declaration on Climate Change (49)	AOSIS	Declaration on Climate Change
Mauritius Strategy (50)	United Nations Educational, Scientific and Cultural Organisation	Addresses problems faced by small island developing states
*Regional*		
Pacific Disaster Risk Reduction and Disaster Management Framework for Action 2005–2015 (51)	United Nations Office for Disaster Risk Reduction	Regional strategy for disaster risk reduction
PIFACC (52)	SPREP	Regional Framework for Action on Climate Change
SPC Climate Change Engagement Strategy (53)	SPC	Framework for SPC's climate change programmes
SPREP strategic action plan (54)	SPREP	Regional climate change action plan
Pacific Regional Action Plan on Sustainable Water Management (55)	South Pacific Applied Geoscience Commission and Asian Development Bank	Sustainable water management in small island countries of pacific
*National*		
Fiji Initial National Communication to UNFCCC (28)	UNFCCC	Fulfil Fiji's obligations to the UNFCCC
People's Charter for Change, Peace and Development (56)	Government of Fiji	Framework for development (five year plan)
Roadmap for Democracy and Sustainable Socio-economic Development (57)	Government of Fiji	Outlines implementation policies to achieve People's Charter
Fiji Strategic Development Plan (58)	Government of Fiji	Plan for identifying and implementing policies
National Strategy and Action Plan under the National Capacity Self-Assessment (59)	Government of Fiji (Department of Environment)	Recommends strategies and programmes for national capacity development (including climate change)
Public Health Act (31)	Government of Fiji	Fijian Constitutional law
National Food and Nutrition Policy (33)	Government of Fiji	Government Policy
Rural Land Use Policy (draft) (34)	Government of Fiji	Government Policy
National Health Emergencies and Disaster Management Plan (HEADMAP) (60)	Government of Fiji (Ministry of Health)	Government Policy

### Content analysis of the NCCP, NCCAS, and related documents

The key document of analysis in this study was the NCCP, which stated that one of its main purposes was to produce guidelines for sectors in considering climate change impacts in planning and development (15). Therefore, it is implied that the policy should provide these guidelines for the health sector and other sectors, in which climate change consequences could impact on human health, such as the agricultural (malnutrition) and water (water-borne disease) sectors.

Directly linked to the NCCP is the NCCAS, a document providing adaptation actions for land-based resources in Fiji (16). The content of both these documents was analysed for the extent to which three of the major health effects of climate change were accounted for, namely, dengue fever, diarrhoeal disease, and nutrition-related illness.

Important supporting documents (including the Public Health Act) were then cross-checked, to analyse the extent to which the impacts of climate change on health were considered in the sector policies and strategies.

#### National Climate Change Policy

*Dengue fever*. Dengue fever was directly mentioned only once in the body of the NCCP, which stated that there would be ‘an increased incidence and severity of vector-borne diseases, for example, dengue fever’. Annex 3 of the NCCP (which expands on climate change impacts, adaptation, and mitigation for each sector) elaborated on the prediction of increased favourable conditions for the dengue fever virus.

Dengue fever was indirectly addressed twice by way of communicable disease strategies: ‘rapid and accurate disease notification’ and ‘strengthening disease surveillance and control systems’. Vector management featured briefly in Annex 3 of the NCCP but not in the main policy body.

*Diarrhoea*.Diarrhoeal illnesses are directly mentioned only once in the main text, where it was stated that food and water-borne diseases, such as diarrhoea, would increase. However, diarrhoea is expanded upon in Annex 3 of the NCCP as a potential health impact of climate change.

The water sector is extensively discussed in the NCCP, and therefore diarrhoea is also indirectly addressed. Compromised food and water sources, impacts on water supply, water contamination, and water infrastructure damage were all listed as predicted impacts.

Annex 3 of the NCCP also mentions recommended human health adaptation activities. These include providing clean water; improving sanitation; and climate-proofing water, health and sanitation infrastructure. Indirect strategies for targeting diarrhoeal disease included in the main policy text are watershed management plans and early warning systems for climate-sensitive disease.

*Malnutrition*. Malnutrition was directly included once within the NCCP, as a foreseeable health problem. Otherwise, food and nutrition were only accounted for indirectly through the agricultural sector, with a focus on decreased sugar and root crop production, which was expanded upon in Annex 3. Compromised food and water resources, along with food security (in terms of production, quality, and nutritional availability and affordability) were also described in detail. Impact on the fisheries was discussed briefly, with a predicted decrease in fish catch volume.

Annex 3 of the NCCP described some adaptation measures for the agriculture and fisheries sectors. Meanwhile, the main policy body outlined two strategies: considering climate change in land-use planning and increasing food security in order to increase resilience.

*Health in general*. Other than the direct and indirect impacts outlined above that were part of the NCCP, there are a small number of further inclusions of the health sector in the document. Objective 1 targets the health sector: ‘review public health … policies to ensure consideration of climate change impact’, while objective 5 recommends improving disaster response and access to public health facilities.

Annex 3 of the NCCP recommends improving responses to public health emergencies caused by extreme events, by strengthening disaster risk reduction and improving disease early warning systems, with a focus on particularly vulnerable areas.

*Cross-sectoral approach*. Throughout the policy, there is an emphasis on the need for a cross-sectoral approach to address the impact of climate change. Indeed, the first objective of the policy is mainstreaming, that is integrating climate change issues in all national and sector policy and planning.

#### National Climate Change Adaptation Strategy

The aim of the NCCAS is to provide adaptation actions for land-based resources, namely, the agricultural, water, forestry, land-use, and biodiversity sectors (16). It is important to note that the health sector was not specifically included as a category in the NCCAS. One of the recommendations of this strategy was to include the health sector in the next version of the NCCAS. This strategy also included a list of current climate change projects taking place in Fiji, while Annex of the NCCP contained sector adaptation matrices for agriculture, water, and land use.

*Dengue fever*. Dengue fever is neither directly nor indirectly accounted for in this strategy.

*Diarrhoea*. Diarrhoea and water-borne diseases are not explicitly accounted for in the NCCAS. Nonetheless, issues within the water sector are extensively covered. Objective 2 recommends upgrading the existing infrastructure and technical capacities, including flood forecasting. Thereafter, the water sector adaptation strategy describes eight methods for improvement. These include enhancing water efficiency, securing water supply, protecting water from pollution, and flood-control measures. A summary of national policies and strategies is also included in the NCCAS for the water sector.

*Malnutrition*. There is no direct mention of malnutrition in this policy. However, the agriculture and land-use sectors, which contribute to the food security of the population, are accounted for in detail. Objective 2 recommends an upgrade of agricultural infrastructure and technical capacities, such as extreme weather forecasting. Seven measures are then listed in the agriculture adaptation plan, including protecting agricultural land, conserving water, and integrating climate change adaptation and disaster risk reduction into agriculture.

An overview of strategies and policies for the agricultural sector are described in the NCCAS. However, it is important to note that no specific policy or action plan exists for the agriculture sector (only a land-use policy).

*Health in general*. Although the health sector is not specifically included in the NCCAS, part of the strategy's vision statement reads as follows, ‘the livelihoods and well-being of the people of Fiji are secured by adapting the land-based resource management sectors to climate change’. It is also acknowledged that the agricultural and water sectors ‘contribute to the general well-being of the people and the country as a whole’.

*Cross-sectoral approach*. Throughout the NCCAS, a cross-sectoral approach is frequently advocated and even included as a specific objective (number 6).

Both the NCCP and the NCCAS strongly advocate a cross-sectoral approach to dealing with climate change. Furthermore, one of the key recommendations of the NCCP is the integration of climate change issues into all national and sectoral policies and planning. Therefore, this study also analysed the relevant sectoral documents in order to assess the extent to which climate change was accounted for.

#### Fiji food and nutrition policy

As previously discussed, malnutrition is predicted to increase as a result of climate change. The Fiji Food and Nutrition Policy neither mentions climate change nor provides a specific strategy to combat its impacts. Indirectly, climate change impacts are addressed, through targeting food access and nutrition-related disease (33).

Improved food security is advocated, with a focus on decreasing the reliance on imports, reducing childhood malnutrition and low birth weight. A reduction in diarrhoea incidence, improving disaster preparedness and monitoring food and nutrition situations are also listed as aims (33).

#### Public health act

Climate change and its projected impacts on human health are not included in the Public Health Act. However, indirectly, they are targeted in various parts of the act, such as (31):

1. Part III: Buildings (which may be impacted by extreme events).
2. Part IV: Food Sale and Distribution (which may affect nutrition-related disease).
3. Part V: Sanitary Services (which may impact diarrhoeal disease).
4. Part VII: Infectious Disease (which may include diarrhoeal disease).
5. Part XI: Mosquitoes (which may impact dengue fever).
6. Part XIII: Water Supply (which may impact diarrhoeal disease).

#### National environmental health action plan

Aligned with the goals of the Fiji Ministry of Health Corporate Plan, the NEHAP provides improvement strategies for water and sanitation; food quality control; vector-borne disease; and environmental health planning and management (32).

#### Rural land use policy for Fiji

Throughout the entire Rural Land Use Policy for Fiji, climate change is not directly accounted for. Ten primary land use issues are analysed and summarised in the policy, with climate change not listed amongst these. Indirectly, strategies to address potential climate change health impacts, such as malnutrition, are included by way of land management practices for food production (34).

#### National water resources policy for the Fiji Islands (draft water policy)

Neither climate change nor its impacts on the water sector and thereby human health are explicitly included in this policy. Indirectly, there is acknowledgement of some events that contribute to poor water quality (and thus increased water-borne disease). Industrial waste and sewage are included as concerns for the sector and there is an overall call for increased protection of water bodies.

Amongst the ten policy goals listed, goal nine indirectly addresses climate change via striving to lessen the impacts of extreme events.

In summary, the NCCP mentions the three health impacts explored in this study mainly implicitly (through water and agricultural sectors). The greatest inclusion of diarrhoea is in Annex 3 of the NCCP rather than the main policy body. The health sector was included as a specific section in the policy and included relevant strategies.

The NCCAS does not specifically include the health sector and, therefore, none of the three investigated health effects are mentioned directly in this strategy. Diarrhoea and malnutrition feature implicitly through the agricultural and water sectors. Finally, none of the four related policies analysed included any acknowledgement of climate change.

## Discussion

This study used a policy analysis approach to understand the extent to which the NCCP and other relevant documents incorporated health responses to climate change in Fiji. In summary, we found that the health impacts of climate change are not adequately accounted for in major relevant policies. We propose in this paper that the content of the policies may have been influenced by the process of the policy development, the context within which the content was formulated and the actors involved.

The process of stakeholder consultation that contributed to the development of these policies would have (logically) been dependent on which actors were involved at the time. Organisations and individuals with the time, resources, and common goals would have been more likely to participate, affecting the policy content. Internal processes, such as communication between the CCU and other departments would have influenced the inclusion and exclusion of content in the final document. Monitoring of the content of the policies would depend on human and financial resources at the time.

Contextual factors make the Fijian population particularly vulnerable to the effects of climate change. Geographically, there are a high number of coastal dwellers, a significant number of people living in poverty and rapid urban migration (with inadequately designed housing). A key opportunity for minimising the harmful effects of rapid, unplanned urbanisation is to ensure that current and future urban development is done in a way that reduces poverty and establishes communities that live within their environment limits (61). Polices in urban planning and urban agriculture help adapt to climate change effects (62). Land-use planning can reduce building in zones at risk of flooding (62).

Implementing early warning systems for extreme events, focussing on vulnerable areas, could reduce the vulnerability of coastal communities. Indeed, this is an important strategy recommended in the NCCP. Annex 3 of the NCCP also recommends improving the emergency response to extreme events (15). This is also critical and could include a cross-sectoral approach organising shelter, transport, food, water, and health care. Food security strategies across sectors, such as food system diversification and urban agricultural policies may be helpful in providing nutritious foods in climate-stressed conditions (61).

The phenomenon of globalisation adds to the pressure on national food security, while brain-drain will affect the capacity of the nation to implement the content of the NCCAS. International sanctions imposed on Fiji after the 2006 Coup affect fiscal resources for policy implementation. The coup also affected the political structure of the Fijian government, which in turn would have had flow on effects for policy development. Policy implementation may need to be adjusted to maintain relevance amongst various culturally diverse communities in Fiji.

There were many actors involved in the NCCP and NCCAS development and continue to be part of its implementation. The enhanced coordination of this diverse representation may allow for a greater alignment of climate change activities across different sectors and organisations. In future, engaging the ministry of health in climate change policy development would be highly beneficial.

In terms of the policy content, it is important that dengue fever diarrhoea and malnutrition be explicitly accounted for in the body of future iterations of the NCCP. In the NCCAS it is stated that the health sector will be accounted for in future editions of the strategy. Human health must be central to future strategies. This would ensure that specific strategies to combat public health effects of climate change are developed.

Throughout both the NCCP and the NCCAS, there is an emphasis on the need for a cross-sectoral approach to address climate change impacts, which aligns with the HiAP approach. This is important, particularly when targeting human health impacts of climate change, as not only is the health sector involved, but also water, sanitation, agriculture, and fisheries. Indeed, the actions of one sector will affect those of another. The NCCP vision of both more effective ‘coordination between administrative and institutional frameworks’ and ‘collaboration amongst government agencies and stakeholders’ is promising.

Including representatives from the health sector in climate change discussions, such as within the CCU, could be one way of increasing cross-sectoral collaboration. The recommended additions to the NCCP and the NCCAS could occur through a policy review process and consultation with the health sector and relevant stakeholders.

Bridging the gap between the NCCP (inclusion of the health sector) and the NCCAS (exclusion of the health sector) is vital. Health professionals have specialised knowledge and skills that can help advocate for preventative action (8). Actions include public health campaigns and advocating local adaptive responses (8). Health care workers can also play critical roles in planning for improved medical access, particularly to vulnerable communities. Strategies for retaining skilled health care workers in Fiji to reduce brain-drain would be of great benefit.

An example of the health sector being incorporated into a climate change adaptation project is the ‘Piloting Climate Change Adaptation to Protect Human Health’ project. This is a partnership among the World Health Organization, Fiji Ministry of Health, and the United Nations Development Programme and includes dengue fever and diarrhoea as priority climate-sensitive diseases. The key objectives are: 1) an early warning system for climate-sensitive disease; 2) improved health sector capacity to respond to climate-sensitive disease; and 3) health adaptation activities in areas of greater risk (30). Important benefits expected are improved community awareness of climate-sensitive diseases and a strengthening of interdisciplinary (within the MoH) and intersectoral collaboration and communication. If effective, results of such projects could also influence future climate change policy.

In summary, neither of the two major climate change policies (the NCCP and NCCAS) consider the health impacts of climate change in detail. In addition, the relevant sectoral documents and policies were also lacking in the extent to which they acknowledged the health impacts of climate change. To benefit public health, it is suggested here that future amendments to the NCCP and NCCAS specifically account for three of the major climate-related health issues explored in this study: dengue fever, malnutrition, and water-borne disease. Engaging the ministry of health in climate change policy development would be highly beneficial. Furthermore, related sectoral strategies in health, water, and agriculture should be revised to include climate change and its influence on human health.

This study could be a useful example for future climate change and health policy analyses in other parts of the world. In particular, applying the HiAP to climate change policy is a useful and innovative approach to strengthen attention paid to the health impacts of climate change.

### Limitations

This study was desk based and reliant on publicly available documents. While this has advantages of reducing bias by analysing information somewhat more objectively, a drawback of this approach is the absence of interviews with stakeholders to supplement and strengthen the contextual knowledge, in particular, in terms of governmental and societal dynamics, such as gaining a deeper understanding of the relationship between the MOH, MOE, and NCCCT. An additional limitation for this study was that some policies were only available in their final draft form. Some documents were not available at all, such as Fiji's second national communication to the UNFCCC (63).

## Conclusion

Dengue fever, malnutrition, and diarrhoeal disease are three of the major climate-related diseases projected to affect Fiji (18). It is vital that all three be specifically included in both the NCCP and the NCCAS to ensure these critical health impacts are at the forefront of planning and development. Including the health sector in future iterations of the NCCAS would broaden the scope of climate-related disease prevention. In addition to addressing the underlying causes of climate-related disease (in sectors such as water and agriculture), the health sector could employ direct prevention and health promoting strategies. Future research could support the Fiji health sector to develop such programmes, as well as identifying the underlying factors contributing to health not being well recognised within the NCCP and NCCAS in Fiji.
